# The Effect of Intensity-Modulated Radiotherapy to the Head and Neck Region on the Oral Innate Immune Response and Oral Microbiome: A Prospective Cohort Study of Head and Neck Tumour Patients

**DOI:** 10.3390/ijms23179594

**Published:** 2022-08-24

**Authors:** Zahra Dorna Mojdami, Abdelahhad Barbour, Morvarid Oveisi, Chunxiang Sun, Noah Fine, Sourav Saha, Cara Marks, Omnia Elebyary, Erin Watson, Howard Tenenbaum, Amir Azarpazhooh, Michael Glogauer

**Affiliations:** 1 Dental Oncology and Maxillofacial Prosthetics Clinic, Princess Margaret Cancer Centre, University Health Network, Toronto, ON M5G 2C1, Canada; 2 Faculty of Dentistry, University of Toronto, Toronto, ON M5G 1X3, Canada; 3 Centre for Advanced Dental Research and Care, Department of Dentistry, Mount Sinai Hospital, Toronto, ON M5G 1X5, Canada

**Keywords:** head and neck cancer, head and neck tumour, oral innate immune response, oral neutrophils, oral polymorphonuclear leukocytes (oPMNs), radiotherapy, radiation therapy, oral microbiome, oral microbiota

## Abstract

Neutrophils, also known as polymorphonuclear leukocytes (PMNs), form a significant component of the innate host response, and the consequence of the interaction between the oral microbiota and PMNs is a crucial determinant of oral health status. The impact of radiation therapy (RT) for head and neck tumour (HNT) treatment on the oral innate immune system, neutrophils in particular, and the oral microbiome has not been thoroughly investigated. Therefore, the objective of this study was to characterize RT-mediated changes in oral neutrophils (oPMNs) and the oral microbiome in patients undergoing RT to treat HNTs. Oral rinse samples were collected prior to, during and post-RT from HNT patients receiving RT at Dental Oncology at Princess Margaret Cancer Centre. The oPMNs counts and activation states were analysed using flow cytometry, and the oral microbiome was analysed using 16S rRNA gene sequencing. Statistically significant (*p* < 0.05) drops in oPMN counts and the activation states of the CD11b, CD16, CD18, CD64 and H3Cit markers from pre-RT to post-RT were observed. Moreover, exposure to RT caused a significant reduction in the relative abundance of commensal Gram-negative bacteria and increased the commensal Gram-positive microbes. Ionizing radiation for the treatment of HNTs simultaneously decreased the recruitment of oPMNs into the oral cavity and suppressed their activation state. The oral microbiome composition post-RT was altered significantly due to RT which may favour the colonization of specific microbial communities unfavourable for the long-term development of a balanced oral microbiome.

## 1. Introduction

Radiotherapy (RT), also known as radiation therapy, is an effective non-surgical tumour treatment modality that has become one of the main methods to treat an array of cancers, including head and neck malignancies [1,2,3]. Head and neck tumours (HNTs) can either be malignant or benign [4] with the most common type of head and neck cancer being epithelial in origin called squamous cell carcinomas (SCCs); its progression includes the accumulation of genetic and epigenetic modifications occurring throughout multiple steps [5]. The main mucosal anatomical locations in which SSCs develop include the sinonasal cavity, the oral cavity, pharynx, larynx and upper oesophagus [6]. Oral complications of RT in the head and neck region include osteoradionecrosis (ORN), oral mucositis (OM) and xerostomia, which have life-altering effects on the quality of life (QoL) of HNT patients [7].

The oral cavity harbours over 700 different species of bacteria that colonize different surfaces [8] and prime/activate oral neutrophils [9]. oPMNs are typically recruited to the oral cavity from the blood circulation, and recently our group has demonstrated that oPMNs activated by oral inflammation can prime systemic innate immune responses [10]. Circulating neutrophils (cPMNs) that are recruited to the oral cavity and become oPMNs, carry out an immune surveillance function and symbiotically interact with commensal microbiota to maintain homeostasis [11]. oPMNs support normal periodontal homeostasis by forming a barrier between the dental biofilm and the oral epithelium. In severe chronic cases of periodontal disease, an increased influx of oPMNs with a hyperactive phenotype will swarm the oral cavity leading to an overactive inflammatory response [12]. Since the function of oral neutrophils is regulated by inflammation and their interaction with the oral microbiome, RT treatment to treat HNTs can either directly or indirectly influence total oral neutrophil counts and activity [13].

Previous studies have demonstrated the functional antimicrobial characteristics of oPMNs, specifically using a periodontitis model, however less attention has been devoted to the basic principles of ionizing radiation and its interaction with the oral microbiome [14,15]. The aim of this study was to describe the effect of ionizing radiation in the form of intensity-modulated radiotherapy (IMRT) during HNT treatment on the quantity and activation states of oral neutrophils and the potential role that oral microbiome plays in these modifications. If RT treatment can induce modifications of oPMNs and the oral microbiome, further investigation into how these changes can influence the development and/or progression of the oral complications associated with RT would be warranted.

## 2. Results

### 2.1. Participants’ Characteristics

A total of 68 patients were recruited through consecutive sampling and completed visit one (pre-RT). Table 1 describes the relevant characteristics of all the patients who completed the pre-RT visit.

A large proportion of patients were ≥65 years of age (48.4%), including 69.1% males. Most patients were diagnosed with pharynx/pharyngeal cancer (59.9%), with the most frequent treatment modality consisting of chemoradiation, irrespective of the type of HNT (38.2%). Of those 31 patients who received chemotherapy (CT), 7 received induction CT and 24 concurrent CT. The average mean dose of radiation to the oral cavity was 3356 cGy.

Samples were collected at pre-RT for 68 patients. Three patients succumbed to their illness and did not complete any further visits after the pre-RT visit. If the participant missed a visit, the participant was not removed from the study and on any follow-up appointments that the participant attended, the oral samples were collected. The reasons for missed visits ranged from participants refusing to participate in that visit due to lack of time or feeling ill, or not showing up for their scheduled appointment.

### 2.2. The Effect of Radiotherapy (RT) on the Absolute Oral Neutrophil (oPMN) Counts

**Figure 1:**
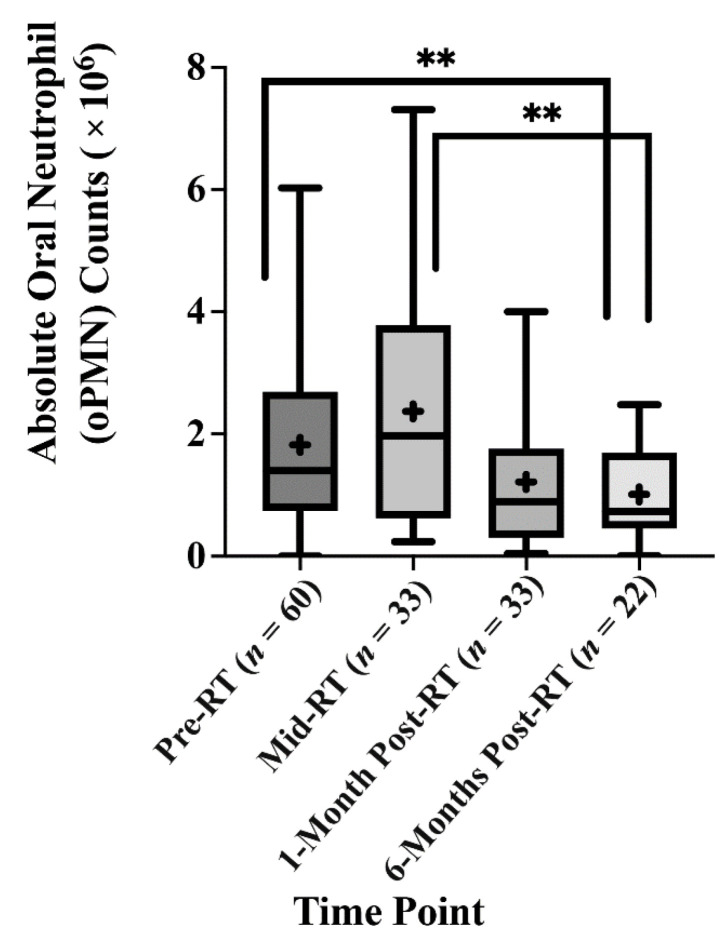
Radiotherapy causes a decrease in the oPMN counts. The absolute counts of the oral neutrophils were analysed across the four-time points. (+) The mean absolute oPMN count. (**) Statistically significant mean difference *p* < 0.005.

One of the main objectives of this study was to determine if RT in HNT patients would alter the neutrophil counts in the oral cavity. Our data showed an increase in the mean absolute oPMN counts in the oral cavity during radiotherapy (Mid-RT) compared to their numbers before radiation (Pre-RT), 1.81 × 10^6^ and 3.71 × 10^6^, respectively, with an adjusted mean difference of 362,000. However, this increase was not statistically significant (*p* = 0.417). There was a statistically significant drop in the mean absolute oPMN counts from mid-RT (3.71 × 10^6^) to 959,277 at six months post-RT, with an adjusted mean difference of 1.457 × 10^6^ (*p* = 0.003). When comparing pre-RT and 6-months post-RT, the mean absolute oPMN counts significantly dropped from 1.81 × 10^6^ at pre-RT to 956,277 at 6-months post-RT, with an adjusted mean difference of 1.096 × 10^6^, *p* = 0.004 (Figure 1) (Table A3). These results indicate that RT plays a vital role in reducing the oPMN numbers recruited to the oral cavity. Furthermore, the oPMN counts at 6-months post-RT did not return to the same counts as seen at pre-RT.

### 2.3. The Effect of Radiotherapy (RT) on the Absolute Oral Neutrophil (oPMN) Marker Counts and Activation States

**Figure 2:**
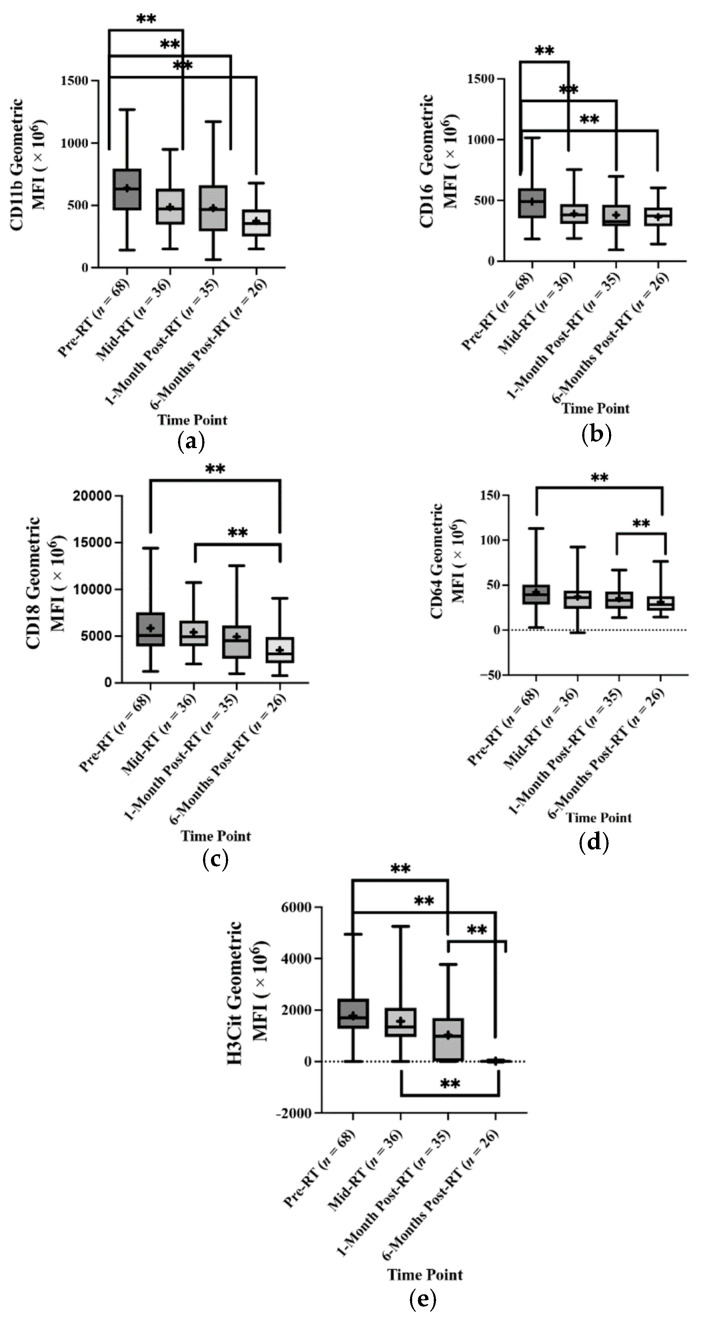
Radiotherapy significantly alters the oral neutrophil (oPMN) activation states. Oral neutrophils collected from HNT patients before, during and post-RT were labelled to analyse CD markers expression. The geometric mean fluorescence intensity (MFI) is shown for CD11b (**a**), CD16 (**b**), CD18 (**c**), CD64 (**d**) and H3Cit (**e**) across the four-time points. (+) The mean geometric MFI. (**) Statistically significant mean difference *p* < 0.005.

oPMN activation is characterized by expression levels of certain cell surface CD markers in the oral cavity depending on the polymicrobial communities they interact with. This study used a panel of seven oral neutrophil CD and H3Cit to evaluate RT’s effect on oPMN priming status and activation. After controlling/adjusting for covariates that have demonstrated effects on absolute oPMN counts within the Linear Mixed Model (LMM) analysis (oral mucositis status (OM), viral load, sex, smoking status, alcohol intake, periodontal disease staging, medications affecting oPMNs and treatment modality), statistically significant drops in the mean geometric MFI for oPMN markers associated with cell migration, adhesion and degranulation were observed. CD11b had a statistically significant drop from 6.38 × 10^8^ at pre-RT to 4.87 × 10^8^ at mid-RT (adjusted mean difference = 24.416 × 10^7^, *p* < 0.001) and 3.74 × 10^8^ at 6-months post-RT (adjusted mean difference = 33.549 × 10^7^, *p* < 0.001). CD16 had a statistically significant drop from 4.91 × 10^8^ at pre-RT to 3.92 × 10^8^ at mid-RT (adjusted mean difference = 90.084 × 10^6^, *p* = 0.040) and 3.64 × 10^8^ at 6-months post-RT (adjusted mean difference = 12.077 × 10^7^, *p* < 0.001). CD18 counts dropped at pre-RT from 5.85 × 10^9^ to 3.51 × 10^9^ with an adjusted mean difference of 26.474 × 10^8^, *p* = 0.001. There was also a significant drop in the CD18 marker counts from mid-RT (5.42 × 10^9^) to 6-months post-RT with an adjusted mean difference of 25.228 × 10^8^, *p* = 0.007. For the CD64 counts, there was a statistically significant reduction in the counts from pre-RT to 6-months RT (3.94 × 10^7^ and 2.956 × 10^7^, respectively) with an adjusted mean count of 13.391 × 10^6^, *p* = 0.001. A statistically significant drop in the CD64 counts was also seen from 1-month post-RT (3.53 × 10^7^) to 6-months post-RT (adjusted mean difference = 10.029 × 10^6^). For the H3Cit counts, statistically significant drops were observed from pre-RT (17.761 × 10^8^) to 1-month post-RT (10.263 × 10^8^) and 6-months post-RT (11.524 × 10^6^) with adjusted mean differences of 95.658 × 10^7^, *p* = 0.001 and 17.000 × 10^8^, *p* < 0.001, respectively. Additionally, statistically significant drops were also seen from mid-RT (15.701 × 10^8^) and 1-month post-RT (adjusted mean difference = 13.498 × 10^8^, *p* = 0.001 and 1-month post-RT to 6-months post-RT (adjusted mean difference = 74.343 × 10^7^, *p* = 0.027 (Figure 2 and Table A7).

**Figure 3:**
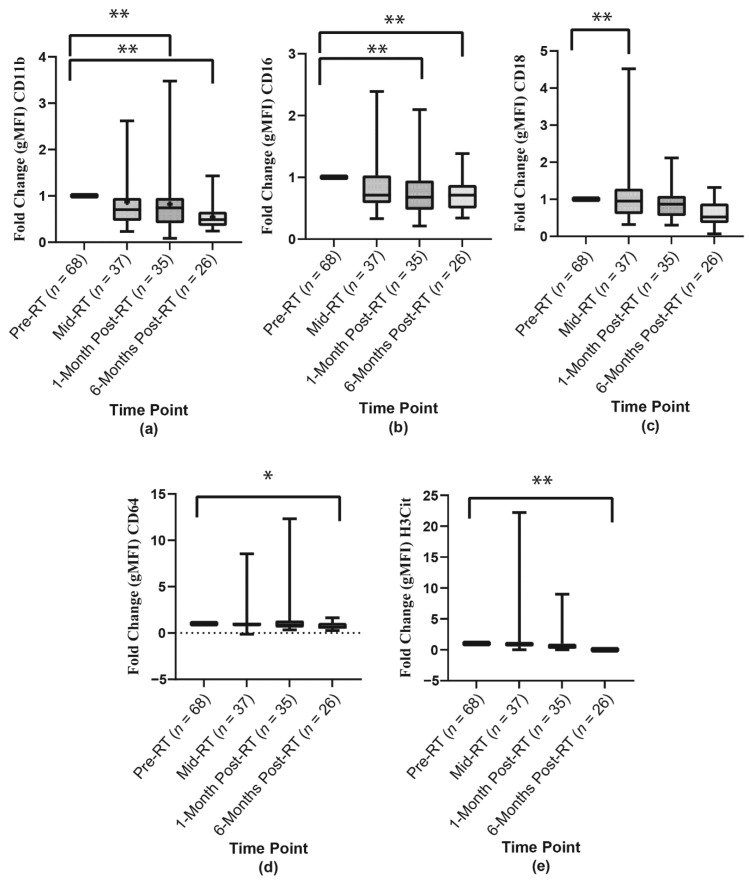
The mean geometric MFI fold-change of the oPMN (**a**) CD11b, (**b**) CD16, (**c**) CD18, (**d**) CD64, and (**e**) H3Cit markers across the four-time points. (+) The mean fold change. (*) Statistically significant mean difference *p* < 0.05. (**) Statistically significant mean difference *p* < 0.005.

To determine the fold change in CD markers expressions, the mean geometric MFI for each oral neutrophil at mid-RT, 1-month post-RT and 6-months post-RT were compared to their baseline (pre-RT). When controlling/adjusting for the covariates; OM status, viral load, sex, smoking status, alcohol intake, periodontal disease staging, medications affecting oPMNs and treatment modality, there were statistically significant fold-change decreases in the levels of CD11b, CD16, CD18, CD64 and H3Cit at the same time points as discussed for the above (Figure 3).

### 2.4. Effect of Radiotherapy (RT) on the Oral Microbiota

**Figure 4:**
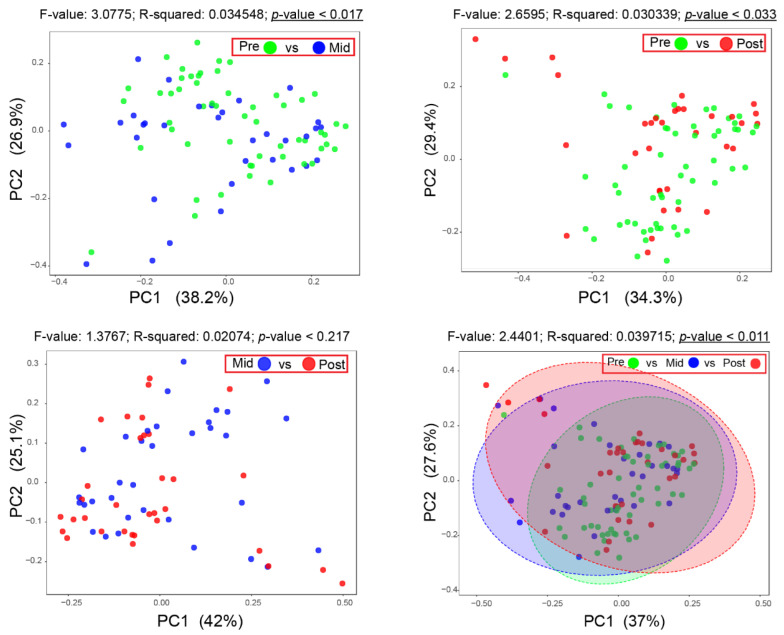
Principle coordinates analysis (PCoA) plots of all oral microbiome samples based on weighted Unifrac distance. Two-dimensional PCoA plots represent the variation between oral microbiome samples in the dataset as follows (Pre-RT vs. Mid-RT, Pre-RT vs. Post-RT, Mid-RT vs. Post-RT and all the three groups Pre-RT vs. Mid-RT vs. Post-RT). Distances between points on the plot represent how similar samples are in terms of microbiota composition and relative abundance. Therefore, points in the plot that are closer in space are more similar in their taxonomic distribution. Significance differences in the *β*-diversity were observed for all tested groups except Mid vs. Post. Underlined *p* values indicate statistical significance (*p* < 0.05). PC1 and PC2 explained 37% and 27% of the variance. Significance was assessed using PERMANOVA.

To examine the effect that RT would have on the oral microbiome of HNT patients, a 16S rRNA gene sequencing analysis of the total bacterial DNA was conducted. After filtering and clustering sequenced reads at 98% identity, the weighted unifrac distance was used to identify changes in the oral microbiome and examine apparent radiation-dependent microbiota changes. Notably, a statistical significance shift (*p* < 0.05) in the *β*-diversity was observed between pre-RT and mid-RT samples, and pre-RT and 1-month post-RT samples. The *β*-diversity was not altered significantly between the samples collected from mid-RT compared to 1-month post-RT time points. These data suggest that RT may directly affect the overall oral microbiome of HNT patients (Figure 4).

**Figure 5:**
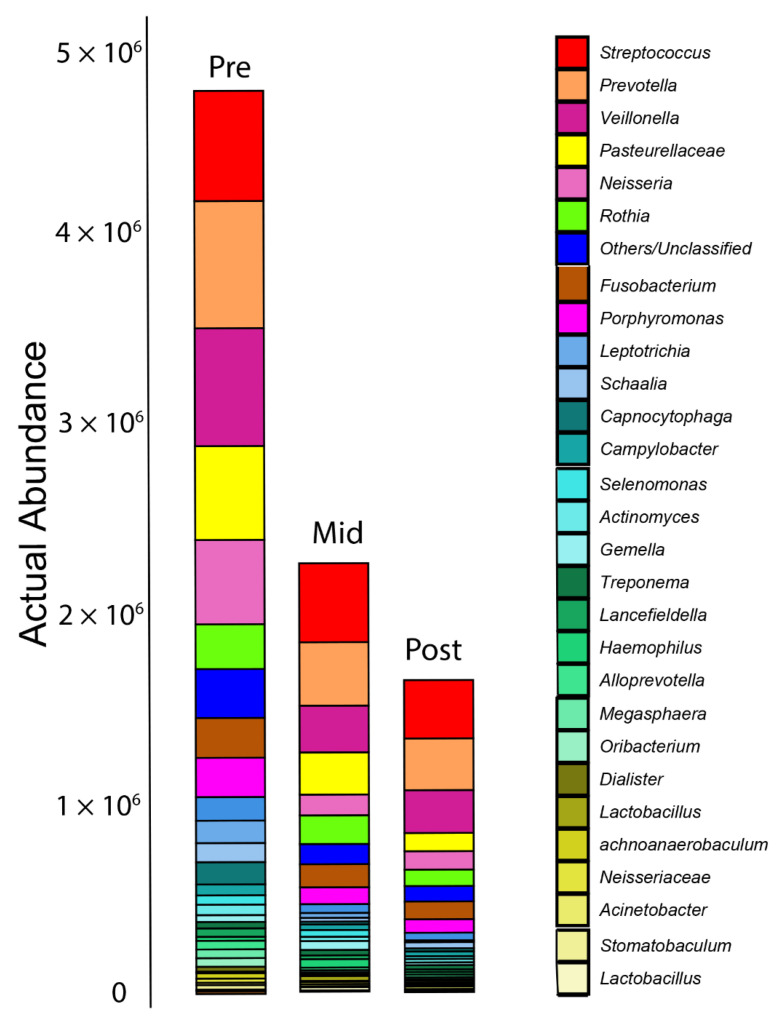
Bar plot for abundance profiling of the oral microbiome. Only the top 30 taxa are shown here as the core bacterial microbiota within the oral cavity. Actual abundance analysis shows a significant reduction in the overall oral microbiome taxa due to radiotherapy.

Actual abundance profiling showed that the overall bacteria numbers were significantly reduced after RT. The bacterial load continued to decline one-month post-RT (Figure 5).

Proportional analysis of each bacterial phylum within the samples showed that *Firmicutes* (containing the most abundant Gram-positive bacteria) were increased by 5–6% within the overall oral microbiome of samples collected after RT (*p* = 0.021). There was a slight increase in *Spirochaetes* (*p* = 0.573) after RT while *Proteobacteria* (*p* = 0.034) were reduced (Table 2).

Importantly, RT did not induce or select for LPS-producing phyla like *Bacteroidetes*, which is usually one of the highest LPS-producing groups in the human microbiome; hence evoking of type I IFN responses by the immune cells in the oral cavity is unlikely to happen during and after RT. At the family and genus levels, a markable increase in numbers of *Streptococcus* occurred at mid-RT. Interestingly, the lactobacilli group, which usually corresponds to dental caries, also increased after RT. Genera that are specifically affected by treatment and reduced after RT include *Veillonella* (mid-RT only), *Haemophilus* (1-month post-only), *Neisseria*, *Actinomyces* (mid-RT only), *Leptotrichia* and *Capnocytophaga* (Table 3).

**Figure 6:**
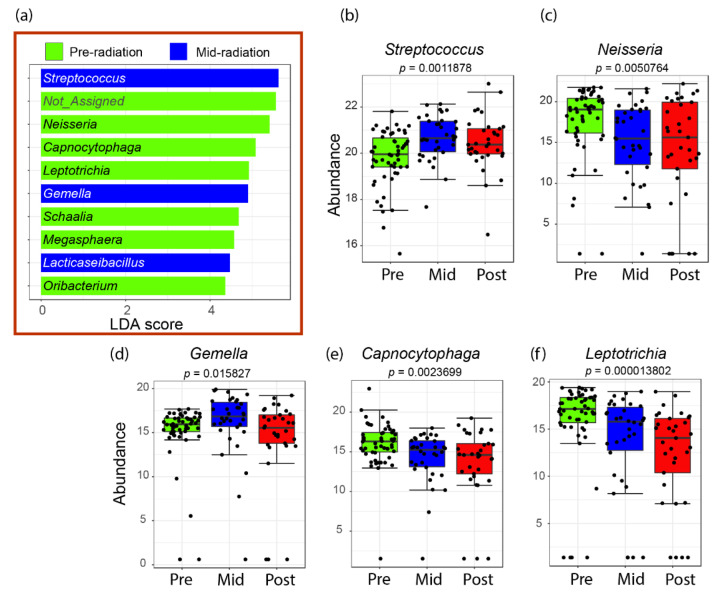
Bacterial genera with increased relative abundance before or after radiotherapy plotted in a LEfSe. The plot bars inside the red box (**a**) correspond to the overall taxa that are significantly abundant either in pre-RT or mid-RT samples. No significant taxa were specified to the post-RT groups since no differences in *β*-diversity were observed between mid and one-month post-radiation. The not assigned bar corresponds to the family *Pasteurellaceae*, of which no specific genus could be identified. Data point visualization is shown here for the top five identified genera, including the mid-RT biomarkers, namely *Streptococcus* (**b**), *Gemella* (**d**) and the top pre-RT taxa *Neisseria* (**c**), *Capnocytophaga* (**e**) and *Leptotrichia* (**f**).

The LDA LEfSe algorithm analysis showed that the streptococci, lactobacilli and the genus *Gemella* are likely the main microbial biomarkers in the oral cavity at the mid-RT point. This agrees with the initial proportional analysis of the relative abundance carried out in this study. LEfSe also showed that before RT, Gram-negative bacteria like *Neisseria*, *Capnocytophaga* and *Leptotrichia* were abundant and declined in the mid and post-RT samples (Figure 6).

## 3. Discussion

Changes in oral neutrophils and oral microbiome can potentially affect the development and severity of oral complications associated with radiation to the head and neck region. Such complications can be acute/short-term or chronic/long-term [16]. Since oPMNs constantly interact with the oral microbiota at the mucosal barrier level [13,17,18], any direct or indirect changes in the polymicrobial community would result in changes in the activation states of oPMNs [19]. Oral microbiome changes due to RT, alteration in oPMN counts, activation and responses to such changes remain poorly understood. A recent study concluded that high blood neutrophil counts are associated with tumours resistant to RT [20]. Lower total neutrophil counts after CR was associated with higher local control rates, metastasis-free survival and overall survival [20]. In our current prospective cohort study, an increase in the absolute counts of oral neutrophils was observed immediately after radiation, followed by a significant decline in the oral neutrophil counts after one-and six months after treatment. This finding may suggest that radiotherapy is likely successful and effective based on what was suggested about the relation between high neutrophil counts and radiotherapy resistance [20].

There have been extensive studies regarding the decrease in peripheral leukocyte counts following RT, and several studies suggest that neutrophils and lymphocytes remain depressed after RT [21,22]. The ionizing radiation may directly damage the oPMNs, thus decreasing their counts in the oral cavity. Radiation’s primary mode of action could explain this, as it depends on breaking the double-stranded DNA of the cells to arrest the cell cycle and promote cell death [23]. The cell response to radiation involves various functions, including reactive oxygen species (ROS)/nitric oxide synthase (NOS) scavenging, DNA damage and repair, signalling evoked by DNA damage and activation of apoptosis, necrosis, necroptosis, saturated autophagy and other pathways of cell death [23]. The radiosensitivity of oPMNs has not been studied intensively; however, studies on circulatory hemopoietic cells have shown that lymphocytes had the greatest sensitivity to radiation, followed by neutrophils and monocytes, then platelets and finally, erythrocytes [24]. It is essential to note that oPMNs have unique functional properties distinct from blood neutrophils or neutrophils in other biological compartments, such as the mucosa or tumour tissue, due to the oral cavity-specific anatomy and physiology [25].

Furthermore, this does not explain decreases in oPMN counts at 1-month and 6-months post-RT as the oPMNs constantly extravasate from the circulation into the oral cavity [17]. A possible biologically plausible explanation is that during RT treatment, as exposure to radiation is repeated and accumulated, there develops constant mucosal inflammation, which explains the increase in oPMN counts at mid-RT. Although this increase is not statistically significant, we predict that a higher sample size would show a statistically significant increase in oral neutrophils at mid-RT. Hou et al. report that constant mucosal inflammation exerts persistent selective pressure for the development of a highly dysbiotic oral microbiome. Therefore, we propose that post-RT, as the effects of radiation wear off, the oral microbial dysbiosis persists and takes over in influencing the changes seen in both the amount and type of oPMNs found in the oral cavity post-RT treatment.

The oPMN CD markers and their surface expression levels can be used for: labelling/defining a specific population of interest, as markers of functionality, and to gauge the state of activation of a particular function [13,26,27]. To our knowledge, this is the first study examining oPMNs and ionizing radiation interactions using oPMN CD marker expression levels to determine oPMN phenotypes.

The changes in expression of specific CD markers on oPMNs may be due to changes in the polymicrobial biofilm present in the oral cavity during and following RT. In the current study, the drop in CD11b, CD18 and CD64 expression by oPMNs indicates a decrease in oPMN adhesion and FcyR, which facilitate the engulfment of IgG-opsonized microbes and trigger cell activation upon cross-linking of several receptors [28]. We have previously demonstrated distinct immune responses of neutrophils when interacting with different oral commensal and pathogenic biofilms. We found that commensal biofilms induced increases in CD66, CD64 and CD55, while pathogenic bacteria induced the expression of CD14 [19,25]. Additionally, commensal biofilms stimulate degranulation, phagocytosis, ROS production and neutrophil extracellular trap (NET) formation, while pathogenic biofilms did not exhibit such effects [19,25].

Whatever the mechanism(s) that may result in the suppression of oPMN function contributes to pathogen persistence and spread [26]. Therefore, post-RT suppression of oPMN function secondary to ionization radiation may be the key to better understanding the development and/or persistence of oral complications associated with RT, specifically OM and ORN.

Previous studies have demonstrated that xerostomia as a result of interruption of salivary flow following RT is correlated with shifts in the oral microbiome resulting in higher abundances of *Streptococcus mutans, Lactobacillus* spp., *Candida* and *Staphylococcus* spp., whereas the number of *S. sanguinis, Neisseria* spp. and *Fusobacterium* spp. tended to decrease [29,30,31]. It has been demonstrated that the progression of OM is directly linked to alterations and dysbiosis of the oral microbiota [32]. Hu et al. found that increasing the dose of ionizing radiation was associated with increased numbers of *Pseudomonas*, *Treponema* and *Granulicatella.* On the other hand, other microbes, including *Prevotella*, *Fusobacterium*, *Leptotrichia*, *Campylobacter*, *Peptostreptococcus* and *Atopobium*, were significantly negatively associated with increased radiation doses [32]. Our current study also found increased levels of *Treponema* (in some patients) and a significant reduction in *Leptotrichia* numbers post-RT; however, our data suggest that *Prevotella* numbers increased after six months post-RT in some patients, but this increase was not significant across all samples tested.

Many studies reported increased streptococci and lactobacilli after RT [33]. Interestingly, a recent study showed that oral microbiota transplantation in mice helped fight head and neck RT-induced OM. As in our current clinical investigation, the study found that RT elevated the abundance of *Streptococcaceae* and *Lactobacillaceae*, which is a shred of direct evidence that RT directly affects the oral microbiota [33].

In summary, our data suggest that radiation may, directly and indirectly, influence the counts and activation states of oPMNs with a shift in oral polymicrobial communities. Oral samples collected during and after radiotherapy showed a significant reduction in the abundance of Gram-negative bacteria and an increase in the genera *Streptococcus*, *Lactobacillus*, *Treponema* and *Prevotella*. This alteration shifted the oral microbiome’s *β*-diversity, positively correlated with reduced oPMN counts and suppressed CD marker activation. These alterations in the oral microbiome and oPMNs did not recover to baseline levels at 6-months post-RT.

## 4. Materials and Methods

### 4.1. Inclusion and Exclusion Criteria of Patients Recruited into this Study

This study consisted of a prospective cohort of adult HNT patients undergoing therapy at our tertiary care institution who presented to the dental oncology and maxillofacial prosthetics clinic for dental assessment and treatment prior to the commencement of RT. Ethics approval was obtained by the University of Toronto and the University Health Network (UHN) (19-5569). The inclusion criteria for this study included adults over the age of 18 who were able to provide informed consent and were diagnosed with a HNT, and RT was included in the course of treatment and who received dental assessments performed at the institutional dental clinic. Exclusion criteria for this study were: pregnant women and patients with immunocompromised conditions/treatments (other than chemotherapy) such as HIV.

### 4.2. Participant Medical Records

Before sample collection, the participants were seen by a dentist at the dental oncology clinic at Princess Margaret Cancer Centre. A complete oral examination and radiographs were completed and taken, respectively. The dentists made oral diagnoses, and information about patients’ relevant dental and health and treatment information was accessed from the patients’ charts at the dental clinic as well as their electronic patient record (EPR) at the University Health Network (UHN), respectively. Periodontal status was carried out using the 2017 classification system [34] based on patients’ radiographs that were interpreted by an Oral Radiologist (GK). All relevant medical information and history were collected from the UHN EPRs for each patient.

### 4.3. Oral Samples Collection

Unstimulated saliva was collected by asking participants to spit into a sterile 50 mL Falcon tube. Next, three separate sterile endodontic paper points (size 30) were used to collect biofilm samples from the dorsum of the tongue and supragingival and subgingival plaque. The paper points were then placed in PowerBead (sand) tube (QIAGEN, Germany). One mL of the collected saliva was added to the biofilm samples (paper points) and stored at −20 °C until future analysis. Patients were asked to rinse with 5 mL of isotonic sodium chloride solution for neutrophil analysis for 30 seconds [13]. Patients then expectorated the rinse sample into a sterile 50 mL falcon tube and were asked to repeat this procedure six times (for a total of 30 mL) with 1 min to 2.5 min intervals between each rinse sample as mentioned previously [13]. The 30 mL oral rinse sample was then placed on ice for no longer than three hours and sent to the lab for processing. Biological samples were collected in the above-mentioned manner during four different time points for each patient: pre-RT, mid-RT, one-month post-RT and six-months post-RT as per patients’ appointment standard of care at the dental oncology clinic at Princess Margaret Cancer Centre.

### 4.4. Quantification of the Absolute Count of oPMNs

Oral rinse samples were fixed with paraformaldehyde (PFA) on ice for 15 min. The cells were harvested at 1000× *g* for 10 min and washed with PBS before storing at 4 °C until further processing. Cell counting of the oPMNs and absolute oral neutrophil counts were found as previously described by Lakschevitz et al. [35].

### 4.5. Flow Cytometry Analysis of the Activation States of Oral Neutrophils (oPMNs)

**Figure A1:**
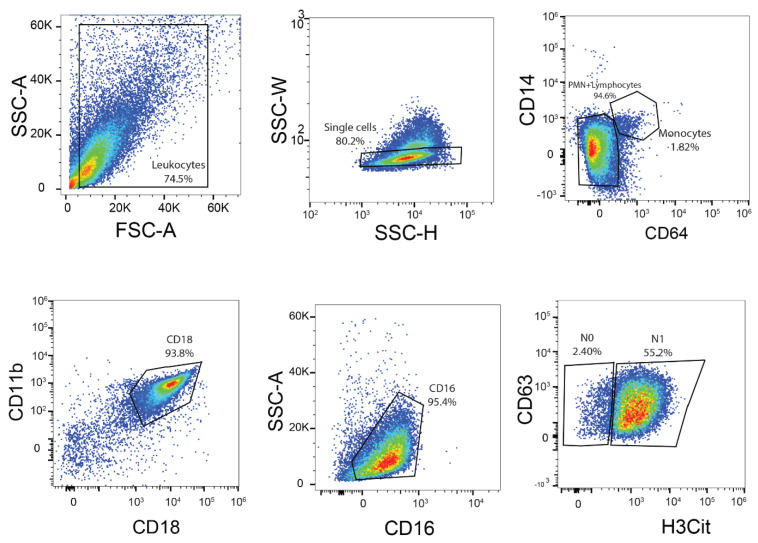
Example of the gating system used for a single patient’s oral rinse sample used in the FlowJo software to measure the geometric MFI for each of the oPMN markers. Gating was performed as follows: Doublets were excluded by SSC-W × SSC-H (side scatter: width × height). To filter out oral macrophages and monocytes, CD14 + area and CD64 + area population were used. the remained population are put on CD11b + area and CD18 + area for reaching PMNs only. To ensure that 100% oral PMNs are captured, a CD16 + area is utilized. The last gating scheme, CD63 + area and H3Cit + area were used to distinguish between primed oPMNs (N1) and regular oPMNs (N0).

The following antibodies were used to label the CD markers: CD16-Alexa Fluor 700 (BioLegend, San Diego, CA, USA), CD63-peridinin chlorophyll protein (PerCP)-Cy5.5 (BioLegend), CD66-allophycocyanin (APC) (eBioscience, Santa Clara, CA, USA), CD14 PE-Cy7 (BioLegend), CD18-brilliant violet 421 (BV421) (BD), CD11b-APC-Cy7 (BioLegend) and CD64-phycoerythrin (PE) (BD). SONY SA3800 flow cytometer was used to acquire the data, and the channel voltages were calibrated manually with rainbow beads to normalize sample acquisition on different days. Compensation was performed with single-stained OneComp eBeads (eBioscience). Data were analysed by FlowJo software (v10; Tree Star), and the geometric mean fluorescence intensities (MFIs) were determined for each CD marker and H3Cit, as mentioned previously by Fine et al. [13]. Gating was then performed, as described by Fine et al. [13] (Figure A1), and the geometric mean fluorescence intensities were used for data analysis.

### 4.6. Oral Microbiome Analysis

DNA extraction was carried out from the oral microbiome samples using DNeasy PowerSoil Pro Kit (Qiagen, Hilden, Germany) according to the manufacturer’s instructions. The DNA concentration and purity were measured with a NanoDrop ND-1000 spectrophotometer (Thermo Fisher Scientific, Waltham, MA, USA). The oral microbiome DNA samples were subjected to 16S rRNA gene sequencing via the Illumina MiSeq platform. First, PCR amplification was carried out by targeting the V4 hypervariable region of the 16S rRNA gene, which was amplified using 515F and 806R primers to allow for multiplexing, as mentioned previously [36]. PCR reactions included 12.5 µL of KAPA2G Robust HotStart ReadyMix (KAPA Biosystems, Wilmington, MA, USA), 1.5 µL of 10 µM forward and reverse primers, 7.5 µL of sterile water and 2 µL of DNA. PCR conditions were 95 °C for 3 min, 22× cycles of 95 °C for 15 s, 50 °C for 15 s and 72 °C for 15 s followed by a 5 min 72 °C extension. The amplified PCR products were then subjected to Illumina MiSeq sequencing according to manufacturer instructions (Illumina, San Diego, CA, USA). Assembled sequences were mapped back to chimera-free denoised sequences at 99% identity operational taxonomic units OTUs, resulting in 69,704 average counts per sample. The mean abundance value was used for the low count filter. The low variance filter was based on standard deviation, which removed 456 low abundance OTUs out of 947 OTUs. OTUs sequences were processed using QIIME [36,37].

### 4.7. Statistical Analyses

For oral neutrophil counts and oral neutrophil CD and H3Cit markers, prior to statistical analyses, outliers in the data were identified and removed using GraphPad Prism Version 9.3.1. To determine significant changes in the absolute oral neutrophil counts, and oral neutrophil markers, linear mixed model (LMM) analyses and adjustments for multiple comparisons were completed using SPSS version 28. All relevant explanatory/covariates were accounted for in the models (Table A2 and Table A6). All the LMM outputs can be found in the Appendix A (Table A1, Table A2, Table A3, Table A4, Table A5, Table A6, Table A7, Table A8, Table A9, Table A10, Table A11 and Table A12). The geometric MFI for each oral neutrophil marker was assessed on the oral neutrophils for each participant mid and post-RT and compared to their baseline to determine the fold-change. LMM analyses were used due to their ability to adjust for imbalances in the number of data points (missing data) [38] across the four-time points. The pairwise comparison using the least significant (LSD) adjustment for multiple comparisons (post-hoc test) was used due to its less restrictive nature with smaller sample sizes. Statistical significance was set at *p* < 0.05.

For the oral microbiome, the beta diversity (*β*-diversity) among sample groups before, during and post-RT was carried out using principal coordinates analysis (PCoA) with weighted Unifrac distance analysis. The Linear Discriminant Analysis (LDA) Effect Size (LEfSe) algorithm was used to identify possible bacterial taxa as biomarkers attributed to each of the treatment groups. Significance was assessed using PERMANOVA.

## 5. Conclusions

Ionizing radiation to the head and neck region to treat HNTs was found to decrease the number of oPMNs as well as suppress oPMN activation through decreases in oPMN markers CD11b, CD16, CD18, CD64 and H3Cit from pre-RT to post-RT. RT also showed a direct effect on the overall microbial load and composition of the oral microbiota of HNT patients. Exposure to RT can cause significant alteration in the microbiome by reducing the relative abundance of commensal Gram-negative microbes and increasing the commensal Gram-positive microbes. While this can be seen as a desired outcome, disturbing the indigenous microbiota composition due to radiation may favour the development of disease-inducing microbial communities and select an abundance of aciduric microbes unfavourable for developing other microbes in the long run. The mechanism as to why these changes occur both in the oral innate immune response and oral microbiota provides an excellent opportunity for further investigation.

The changes in the oral innate immune response and oral microbiome as a result of ionizing radiation to treat HNTs provide the opportunity to determine how these changes may affect and contribute to oral complications commonly seen in patients undergoing RT. In the case of a significant shift in the oral microbiome, more frequent preventive periodontal oral health care and follow-up may be required, and administration of oral probiotics to counteract the RT changes in the oral microbiota to improve the oral health outcomes of patients post-RT may be recommended. Furthermore, targeted dental treatments and preventive measures for this cohort of patients would need to be incorporated into their standard of care to assist in maintaining excellent oral health and function.

## Figures and Tables

**Table 1:** Relevant characteristics of the participants.

Characteristic	*N*	Percentage %
**Type of HNT**		
Pharynx	38	55.88
Larynx	7	10.29
Sinus	1	1.47
Salivary	2	2.94
Oral	12	17.65
Other	6	8.82
Benign	2	2.94
**Total**	68	100.00
**Treatment Type/Group/Modality**		
Radiation	21	30.88
Chemoradiation	26	38.24
Surgery and Adjuvant Radiation	16	23.53
Surgery and Adjuvant Chemoradiation	5	7.35
**Total**	68	100.00
**Type of Chemotherapy**		
Induction	7	22.60
Concurrent	24	77.42
**Total**	31	100.00

**Table 2:** The relative abundance profile (Phylum level) of the total oral microbiome at three different time points.

Phylum	Pre-Radiation	Mid-Radiation	Post-Radiation
*Firmicutes*	35.08%	40.18%	40.98%
*Bacteroidetes*	22.51%	20.5%	23.43%
*Proteobacteria*	21.82%	18.96%	15.13%
*Actinobacteria*	9.71%	10.62%	10.13%
*Fusobacteria*	7.93%	6.3%	6.18%
*Campilobacterota*	1.22%	1.33%	1.57%
*Spirochaetes*	0.71%	1.26%	1.35%

**Table 3:** Relative abundance profile (Family level) of the total oral microbiome with changes at the three different time points.

Family	Most Abundant Genus	Pre	Mid	Post
*Prevotellaceae*	*Prevotella*	15.09%	15.52%	17.36%
*Veillonellaceae*	*Veillonella*	15.07%	12.11%	15.42%
*Streptococcaceae*	*Streptococcus*	12.2%	18.45%	18.74%
*Pasteurellaceae*	*Haemophilus* and others	10.83%	11.74%	6.68%
*Neisseriaceae*	*Neisseria*	9.87%	5.56%	6.38%
*Micrococcaceae*	*Rothia*	4.97%	6.74%	5.28%
*Fusobacteriaceae*	*Fusobacterium*	4.32%	3.94%	4.34%
*Actinomycetaceae*	*Actinomyces, Schaalia*	3.29%	1.91%	3.11%
*Porphyromonadaceae*	*Porphyromonas, Tannerella*	2.85%	2.33%	2.82%
*Leptotrichiaceae*	*Leptotrichia*	2.69%	1.27%	0.68%
*Flavobacteriaceae*	*Capnocytophaga*	2.49%	0.63%	0.98%
*Lachnospiraceae*	*Stomatobaculum*, *Oribacterium*	2.45%	1.11%	1.29%
*Selenomonadaceae*	*Selenomonas*	1.3%	1.8%	0.93%
*Campylobacteraceae*	*Campylobacter*	1.22%	1.33%	1.57%
*Atopobiaceae*	*Lancefieldella*	0.98%	0.92%	0.89%
*Bacillales_Incertae_Sedis_XI*	*Gemella*	0.75%	2.06%	0.87%
*Spirochaetaceae*	*Treponema*	0.71%	1.26%	1.35%
*Moraxellaceae*	*Acinetobacter*	0.44%	0.61%	1.07%
*Lactobacillaceae*	*Lactobacillus*, *Lacticaseibacillus*	0.38%	2.4%	1.53%

## Data Availability

The data presented in this study are available in the Appendix A of this paper.

## Appendix A

**Table A1:** Absolute oPMN counts and descriptive statistics across the four time points.

Absolute oPMN Counts	*N*	Minimum	Maximum	Mean	Std. Deviation
Pre-Radiation	60	1880	6.03 × 10^6^	1.81 × 10^6^	1.45 × 10^6^
Mid-Radiation	33	238,560	46.5 × 10^6^	3.71 × 10^6^	7.91 × 10^6^
1-Month Post-Radiation	33	41,700	4.54 × 10^6^	1.28 × 10^6^	1.19 × 10^6^
6-Months Post-Radiation	22	6280	2.48 × 10^6^	959,277	709,390

**Table A2:** Results of the LMM analysis adjusting for covariates on the mean oPMN absolute counts across the four time points.

Source	Numerator df	Denominator df	F	Sig.
Mean Absolute oPMN Counts				
Intercept	1	12.645	8.283	0.013
Time	3	40.164	4.389	**0.009 ***
Mucositis	1	46.917	0.233	0.632
Viral Load	1	10.444	0.054	0.821
Sex	1	11.008	1.177	0.301
Smoking Status	2	12.350	1.607	0.240
Alcohol Intake	2	11.025	0.564	0.584
Periodontal Disease	3	11.284	1.920	0.183
Medications Affecting oPMNs	1	11.673	0.022	0.884
Treatment Modality	2	11.654	1.156	0.348

* Statistically significant at *p* < 0.05.

**Table A3:** Pairwise comparison of the mean oPMN counts across the four time points.

Time (a)	Time (b)	Adjusted Mean Difference (a–b)	Std. Error	df	Sig. ^a^	95% Confidence Interval	
						Lower Bound	Upper Bound
Mean Absolute oPMN Count							
Pre-Radiation	Mid-Radiation	−362,000	441,000	41.483	0.417	1.252 × 10^6^	529,000
Pre-Radiation	1-Month Post-Radiation	307,000	366,000	39.176	0.407	−434,000	1.047 × 10^6^
Pre-Radiation	6-Months Post-Radiation	**1.096 × 10^6^ ***	357,000	38.757	**0.004 ***	373,000	1.819 × 10^6^
Mid-Radiation	1-Month Post-Radiation	668,000	463,000	43.963	0.156	−266,000	1.602 × 10^6^
Mid-Radiation	6-Months Post-Radiation	**1.457 × 10^6^ ***	468,000	40.591	**0.003 ***	513,000	2.402 × 10^6^
1-Month Post-Radiation	6-Months Post-Radiation	789,000	400,000	38.636	0.056	−21,000	1.599 × 10^6^

* Statistically Significant at *p* < 0.05. ^a^ Adjustment for multiple comparisons: Least Significant Difference (LSD).

**Table A4:** The fixed effect estimates of the mean oPMN absolute counts across the four time points.

Parameter	Estimate	Std. Error	df	t	Sig.	95% Confidence Interval	
						Lower Bound	Upper Bound
Mean Absolute oPMN Count							
**Parameter**							
**Intercept**	8.272 × 10^6^	4.162 × 10^6^	12.045	1.988	0.070	−792,000	17.336 × 10^6^
**Time**							
Pre-Radiation	0	0					
Mid-Radiation	362,000	441,000	41.483	0.819	0.417	−529,000	1.252 × 10^6^
1-Month Post-Radiation	−307,000	366,000	39.176	−0.837	0.407	−1.047 × 10^6^	434,000
6-Months Post-Radiation	**−1.096 × 10^6^ ***	357,000	38.757	−3.066	**0.004 ***	−1.819 × 10^6^	−373,000

* Statistically Significant at *p* < 0.05.

**Table A5:** oPMN marker counts and descriptive statistics.

	*N*	Minimum	Maximum	Mean	Std. Deviation
**CD11b Counts**					
Pre-Radiation	67	1.42 × 10^8^	1.27 × 10^9^	6.38 × 10^8^	2.31 × 10^8^
Mid-Radiation	37	1.51 × 10^8^	9.50 × 10^8^	4.87 × 10^8^	1.94 × 10^6^
1-Month Post-Radiation	35	6.55 × 10^7^	1.17 × 10^9^	4.79 × 10^8^	2.47 × 10^8^
6-Months Post-Radiation	26	1.50 × 10^8^	6.79 × 10^8^	3.74 × 10^8^	1.48 × 10^8^
**CD14 Counts**					
Pre-Radiation	61	0.00	1.33 × 10^8^	7.37 × 10^7^	2.71 × 10^7^
Mid-Radiation	37	0.00	1.85 × 10^8^	6.73 × 10^7^	4.12 × 10^7^
1-Month Post-Radiation	35	0.00	2.32 × 10^8^	7.91 × 10^7^	5.24 × 10^7^
6-Months Post-Radiation	25	−3.26 × 10^7^	3.86 × 10^8^	9.45 × 10^7^	1.14 × 10^8^
**CD16 Counts**					
Pre-Radiation	68	1.84 × 10^8^	1.02 × 10^9^	4.91 × 10^8^	1.64 × 10^8^
Mid-Radiation	36	1.88 × 10^8^	7.55 × 10^8^	3.92 × 10^8^	1.15 × 10^8^
1-Month Post-Radiation	35	9.42 × 10^7^	6.98 × 10^8^	3.81 × 10^8^	1.46 × 10^8^
6-Months Post-Radiation	26	1.43 × 10^8^	6.05 × 10^8^	3.64 × 10^8^	1.17 × 10^8^
**CD18 Counts**					
Pre-Radiation	67	1.23 × 10^9^	1.44 × 10^10^	5.85 × 10^9^	2.85 × 10^9^
Mid-Radiation	37	2.02 × 10^9^	1.07 × 10^10^	5.42 × 10^9^	2.00 × 10^9^
1-Month Post-Radiation	35	9.95 × 10^8^	1.25 × 10^10^	4.92 × 10^9^	2.77 × 10^9^
6-Months Post-Radiation	25	7.74 × 10^8^	9.05 × 10^9^	3.51 × 10^9^	1.93 × 10^9^
**CD63 Counts**					
Pre-Radiation	66	8.90 × 10^7^	4.86 × 10^8^	2.44 × 10^8^	8.29 × 10^7^
Mid-Radiation	37	9.22 × 10^7^	5.50 × 10^8^	2.66 × 10^8^	1.04 × 10^8^
1-Month Post-Radiation	35	6.28 × 10^7^	6.12 × 10^8^	2.55 × 10^8^	1.25 × 10^8^
6-Months Post-Radiation	26	7.56 × 10^8^	4.23 × 10^8^	2.19 × 10^8^	9.05 × 10^7^
**CD64 Counts**					
Pre-Radiation	64	2.80 × 10^6^	8.39 × 10^7^	3.94 × 10^7^	1.62 × 10^7^
Mid-Radiation	36	−2.81 × 10^6^ *	7.20 × 10^7^	3.55 × 10^7^	1.53 × 10^7^
1-Month Post-Radiation	35	1.38 × 10^7^	6.69 × 10^7^	3.53 × 10^7^	1.31 × 10^7^
6-Months Post-Radiation	25	1.45 × 10^7^	5.3 × 10^7^	2.956 × 10^7^	1.02 × 10^7^
**CD66a Counts**					
Pre-Radiation	68	6.10 × 10^7^	7.05 × 10^8^	4.01 × 10^8^	1.45 × 10^8^
Mid-Radiation	37	8.35 × 10^7^	7.00 × 10^8^	3.75 × 10^8^	1.46 × 10^8^
1-Month Post-Radiation	35	1.46 × 10^8^	9.26 × 10^8^	3.76 × 10^8^	1.67 × 10^8^
6-Months Post-Radiation	26	3.92 × 10^7^	5.64 × 10^8^	3.34 × 10^8^	1.40 × 10^8^
**H3Cit Counts**					
Pre-Radiation	67	0.00	4.94 × 10^9^	17.761 × 10^8^	1.05 × 10^9^
Mid-Radiation	37	0.00	5.25 × 10^9^	15.701 × 10^8^	1.23 × 10^9^
1-Month Post-Radiation	34	0.00	3.77 × 10^9^	10.263 × 10^8^	9.41 × 10^8^
6-Months Post-Radiation	22	−9.13 × 10^6^	49.70 × 10^6^	11.524 × 10^6^	14.222 × 10^6^

* A negative value (MFI) = 0.00.

**Table A6:** Results of the LMM analyses adjusting for covariates for oPMN markers CD11b, CD16, CD18 CD64 and H3Cit.

Source	Numerator df	Denominator df	F	Sig.
**CD11b Counts**				
Intercept	1	14.054	11.923	0.004
Time	3	48.407	16.457	**<0.001 ***
Mucositis	1	55.651	0.538	0.467
Viral Load	1	10.833	4.266	0.064
Sex	1	11.135	0.104	0.753
Smoking Status	2	13.852	0.398	0.679
Alcohol Intake	2	11.322	0.622	0.554
Periodontal Disease	3	11.241	1.086	0.395
Medications Affecting oPMNs	1	13.176	1.776	0.205
Treatment Modality	3	13.185	1.116	0.378
**CD16 Counts**				
Intercept	1	11.935	13.760	0.003
Time	3	45.527	5.685	**0.002 ***
Mucositis	1	51.411	1.067	0.306
Viral Load	1	10.321	1.494	0.249
Sex	1	10.505	0.575	0.465
Smoking Status	2	11.971	0.260	0.775
Alcohol Intake	2	10.547	0.031	0.970
Periodontal Disease	3	10.664	0.120	0.946
Medications Affecting oPMNs	1	11.722	0.169	0.689
Treatment Modality	3	11.472	0.988	0.433
**CD18 Counts**				
Intercept	1	13.635	6.599	0.023
Time	3	47.030	5.205	**0.003 ***
Mucositis	1	53.979	3.020	0.088
Viral Load	1	11.312	0.000	0.999
Sex	1	11.184	1.761	0.211
Smoking Status	2	13.599	0.913	0.424
Alcohol Intake	2	11.423	1.430	0.279
Periodontal Disease	3	11.399	2.160	0.149
Medications Affecting oPMNs	1	13.062	0.058	0.813
Treatment Modality	3	12.943	0.822	0.505
**CD64 Counts**				
Intercept	1	12.040	9.214	0.010
Time	3	44.325	4.035	**0.013 ***
Mucositis	1	52.703	0.097	0.756
Viral Load	1	10.127	0.864	0.374
Sex	1	10.519	0.004	0.949
Smoking Status	2	12.228	0.356	0.707
Alcohol Intake	2	10.491	0.343	0.718
Periodontal Disease	3	10.810	0.353	0.788
Medications Affecting oPMNs	1	11.888	0.057	0.815
Treatment Modality	3	11.524	0.348	0.792
**H3Cit Counts**				
Intercept	1	12.833	0.216	0.650
Time	3	44.412	12.332	**<0.001 ***
Mucositis	1	50.168	0.009	0.924
Viral Load	1	11.381	2.567	0.136
Sex	1	11.125	0.012	0.915
Smoking Status	2	12.730	0.145	0.866
Alcohol Intake	2	11.525	0.678	0.527
Periodontal Disease	3	11.399	0.119	0.947
Medications Affecting oPMNs	1	12.438	1.596	0.230
Treatment Modality	3	12.245	0.079	0.970

* Statistically significant at *p* < 0.05.

**Table A7:** Pairwise comparisons of the mean difference in the oPMN CD marker counts and H3Cit counts.

Time (a)	Time (b)	Adjusted Mean Difference (a–b)	Std. Error	df	Sig. ^a^	95% Confidence Interval	
						Lower Bound	Upper Bound
**CD11b Counts**							
Pre-Radiation	Mid-Radiation	**24.416 × 10^7^ ***	62.180 × 10^6^	50.624	**0.000 ***	11.930 × 10^7^	36.901 × 10^7^
Pre-Radiation	1-Month Post-Radiation	**25.332 × 10^7^ ***	52.405 × 10^6^	47.250	**0.000 ***	14.791 × 10^7^	35.873 × 10^7^
Pre-Radiation	6-Months Post-Radiation	**33.549 × 10^7^ ***	51.973 × 10^6^	47.280	**0.000 ***	23.095 × 10^7^	44.003 × 10^7^
Mid-Radiation	1-Month Post-Radiation	9.163 × 10^6^	63.617 × 10^6^	51.309	0.886	−11.853 × 10^7^	13.686 × 10^7^
Mid-Radiation	6-Months Post-Radiation	91.330 × 10^6^	66.535 × 10^6^	47.778	0.176	−42.465 × 10^6^	22.512 × 10^7^
1-Month Post-Radiation	6-Months Post-Radiation	82.166 × 10^6^	57.581 × 106	46.363	0.160	−33.713 × 10^6^	19.805 × 10^7^
**CD14 Counts**							
Pre-Radiation	Mid-Radiation	13.781 × 10^6^	22.577 × 10^6^	50.957	0.544	−31.546 × 10^6^	59.107 × 10^6^
Pre-Radiation	1-Month Post-Radiation	5.998 × 10^6^	19.551 × 10^6^	47.002	0.760	−33.334 × 10^6^	45.330 × 10^6^
Pre-Radiation	6-Months Post-Radiation	−14.328 × 10^6^	19.684 × 10^6^	47.747	0.470	−53.911 × 10^6^	25.254 × 10^6^
Mid-Radiation	1-Month Post-Radiation	−7.783 × 10^6^	22.900 × 10^6^	53.140	0.735	−53.712 × 10^6^	38.147 × 10^6^
Mid-Radiation	6-Months Post-Radiation	−28.109 × 10^6^	24.730 × 10^6^	48.562	0.261	−77.817 × 10^6^	21.599 × 10^6^
1-Month Post-Radiation	6-Months Post-Radiation	−20.326 × 10^6^	21.720 × 10^6^	47.053	0.354	−64.020 × 10^6^	23.368 × 10^6^
**CD16 Counts**							
Pre-Radiation	Mid-Radiation	**90.084 × 10^6^ ***	42.543 × 10^6^	47.090	**0.040 ***	4.502 × 10^6^	17.567 × 10^7^
Pre-Radiation	1-Month Post-Radiation	**12.191 × 10^7^ ***	35.471 × 10^6^	44.793	**0.001 ***	50.456 × 10^6^	19.336 × 10^7^
Pre-Radiation	6-Months Post-Radiation	**12.077 × 10^7^ ***	35.181 × 10^6^	44.811	**0.001 ***	49.902 × 10^6^	19.164 × 10^7^
Mid-Radiation	1-Month Post-Radiation	31.823 × 10^6^	43.622 × 10^6^	47.496	0.469	−55.908 × 10^6^	11.956 × 10^7^
Mid-Radiation	6-Months Post-Radiation	30.684 × 10^6^	45.108 × 10^6^	45.150	0.500	−60.160 × 10^6^	12.153 × 10^7^
1-Month Post-Radiation	6-Months Post-Radiation	−1.139 × 10^6^	38.872 × 10^6^	44.224	0.977	−79.468 × 10^6^	77.191 × 10^6^
**CD18 Counts**							
Pre-Radiation	Mid-Radiation	12.462 × 107	82.916 × 10^7^	48.845	0.881	−15.418 × 10^8^	17.910 × 10^8^
Pre-Radiation	1-Month Post-Radiation	11.059 × 10^8^	69.625 × 10^7^	45.806	0.119	−29.575 × 10^7^	25.075 × 10^8^
Pre-Radiation	6-Months Post-Radiation	**26.474 × 10^8^ ***	70.782 × 10^7^	46.379	**0.001 ***	12.230 × 10^8^	40.719 × 10^8^
Mid-Radiation	1-Month Post-Radiation	98.127 × 10^7^	84.894 × 10^7^	49.430	0.253	−72.436 × 10^7^	26.869 × 10^8^
Mid-Radiation	6-Months Post-Radiation	**25.2282 × 10^8^ ***	89.446 × 10^7^	46.299	**0.007 ***	72.267 × 10^7^	43.230 × 10^8^
1-Month Post-Radiation	6-Months Post-Radiation	15.4155 × 10^8^	78.019 × 10^7^	45.514	0.054	−29.339 × 10^6^	31.124 × 10^8^
**CD63 Counts**							
Pre-Radiation	Mid-Radiation	−15.487 × 10^6^	29.691 × 10^6^	46.880	0.604	−75.222 × 10^6^	44.248 × 10^6^
Pre-Radiation	1-Month Post-Radiation	2.840 × 10^6^	25.243 × 10^6^	44.809	0.911	−48.009 × 10^6^	53.689 × 10^6^
Pre-Radiation	6-Months Post-Radiation	23.222 × 10^6^	24.822 × 10^6^	44.676	0.355	−6.782 × 10^6^	73.226 × 10^6^
Mid-Radiation	1-Month Post-Radiation	18.327 × 10^6^	30.004 × 10^6^	48.052	0.544	−42.000 × 10^6^	78.653 × 10^6^
Mid-Radiation	6-Months Post-Radiation	38.709 × 10^6^	31.317 × 10^6^	44.611	0.223	−24.383 × 10^6^	10.180 × 10^7^
1-Month Post-Radiation	6-Months Post-Radiation	20.382 × 10^6^	27.061 × 10^6^	43.738	0.455	−34.165 × 10^6^	74.930 × 10^6^
**CD64 Counts**							
Pre-Radiation	Mid-Radiation	2.862 × 10^6^	4.926 × 10^6^	46.091	0.564	−7.052 × 10^6^	12.777 × 10^6^
Pre-Radiation	1-Month Post-Radiation	3.362 × 10^6^	3.988 × 10^6^	43.284	0.404	−4.679 × 10^6^	11.402 × 10^6^
Pre-Radiation	6-Months Post-Radiation	**13.391 × 10^6^ ***	3.924 × 10^6^	43.031	**0.001 ***	5.478 × 10^6^	21.304 × 10^6^
Mid-Radiation	1-Month Post-Radiation	499,000	5.003 × 10^6^	46.814	0.921	−9.567 × 10^6^	10.566 × 10^6^
Mid-Radiation	6-Months Post-Radiation	10.529 × 10^6^	5.237 × 10^6^	44.627	0.050	−21,000	21.079 × 10^6^
1-Month Post-Radiation	6-Months Post-Radiation	**10.029 × 10^6^ ***	4.339 × 10^6^	42.929	**0.026 ***	1.279 × 10^6^	18.780 × 10^6^
**CD66a Counts**							
Pre-Radiation	Mid-Radiation	95.612 × 10^6^	59.322 × 10^6^	34.123	0.116	−24.930 × 10^6^	21.615 × 10^7^
Pre-Radiation	1-Month Post-Radiation	57.626 × 10^6^	55.213 × 10^6^	56.989	0.301	−52.937 × 10^6^	16.819 × 10^7^
Pre-Radiation	6-Months Post-Radiation	65.729 × 10^6^	54.033 × 10^6^	40.912	0.231	−43.400 × 10^6^	17.486 × 10^6^
Mid-Radiation	1-Month Post-Radiation	−37.986 × 10^6^	59.696 × 10^6^	47.322	0.528	−15.806 × 10^7^	82.084 × 10^6^
Mid-Radiation	6-Months Post-Radiation	−29.883 × 10^6^	67.804 × 10^6^	33.682	0.662	−16.773 × 10^7^	10.796 × 10^7^
1-Month Post-Radiation	6-Months Post-Radiation	8.103 × 10^6^	61.551 × 10^6^	49.889	0.896	−11.553 × 10^7^	13.174 × 10^7^
**H3Cit Counts**							
Pre-Radiation	Mid-Radiation	35.022 × 10^7^	33.822 × 10^7^	45.854	0.306	−33.065 × 10^7^	10.311 × 10^8^
Pre-Radiation	1-Month Post-Radiation	**95.658 × 10^7^ ***	28.177 × 10^7^	43.628	**0.001 ***	38.858 × 10^7^	15.246 × 10^8^
Pre-Radiation	6-Months Post-Radiation	**17.000 × 10^8^**	29.568 × 10^7^	43.604	**0.000 ***	11.0396 × 10^8^	22.961 × 10^8^
Mid-Radiation	1-Month Post-Radiation	60.636 × 10^7^	34.695 × 10^7^	46.357	0.087	−1.863 × 10^6^	13.0459 × 10^8^
Mid-Radiation	6-Months Post-Radiation	**13.498 × 10^8^ ***	366.446	43.747	**0.001 ***	61.115 × 10^7^	20.884 × 10^8^
1-Month Post-Radiation	6-Months Post-Radiation	**74.343 × 10^7^ ***	32.457 × 10^7^	43.541	**0.027 ***	89.098 × 10^6^	13.978 × 10^8^

* Statistically Significant at *p* < 0.05. ^a^ Adjustment for multiple comparisons: Least Significant Difference (LSD).

**Table A8:** The Fixed effect estimates for the oPMN CD markers and H3Cit marker.

Parameter	Estimate	Std. Error	df	t	Sig. ^a^	95% Confidence Interval	
						Lower Bound	Upper Bound
**CD11b**							
**Intercept**	45.377 × 10^7^	31.628 × 10^7^	13.748	1.435	0.174	−22.575 × 10^7^	11.333 × 10^8^
Pre-Radiation	0	0					
Mid-Radiation	**−24.416 × 10^7^ ***	62.180 × 10^6^	50.624	−3.927	**0.000 ***	−36.901 × 10^7^	−11.930 × 10^7^
1-Month Post-Radiation	**−25.332 × 10^7^ ***	52.405 × 10^6^	47.250	−4.834	**0.000 ***	−35.873 × 10^7^	−14.791 × 10^7^
6-Months Post-Radiation	**−33.549 × 10^7^**	51.973 × 10^6^	47.280	−6.455	**0.000 ***	−44.003 × 10^7^	−23.095 × 10^7^
**CD14**							
**Intercept**	94.695 × 10^6^	85.770 × 10^6^	14.102	1.104	0.288	−89.137 × 10^6^	27.853 × 10^7^
Pre-Radiation	0	0					
Mid-Radiation	−13.781 × 10^6^	22.577 × 10^6^	50.957	−0.610	0.544	−59.107 × 10^6^	31.546 × 10^6^
1-Month Post-Radiation	−5.998 × 10^6^	19.551 × 10^6^	47.002	−0.307	0.760	−45.330 × 10^6^	33.334 × 10^6^
6-Months Post-Radiation	14.328 × 10^6^	19.684 × 10^6^	47.747	0.728	0.470	−25.254 × 10^6^	53.911 × 10^6^
**CD16**							
**Intercept**	31.485 × 10^7^	28.812 × 10^7^	11.747	1.093	0.296	−31.440 × 10^7^	94.411 × 10^7^
Pre-Radiation	0	0					
Mid-Radiation	**−90.084 × 10^6^ ***	42.543 × 10^6^	47.090	−2.117	**0.040 ***	−17.567 × 10^7^	−4.502 × 10^6^
1-Month Post-Radiation	**−12.191 × 10^7^ ***	35.471 × 10^6^	44.793	−3.437	**0.001 ***	−19.336 × 10^7^	−50.456 × 10^6^
6-Months Post-Radiation	**−12.077 × 10^7^ ***	35.181 × 10^6^	44.811	−3.433	**0.001 ***	−19.164 × 10^7^	−49.902 × 10^6^
**CD18**							
**Intercept**	30.963 × 10^8^	45.294 × 10^8^	13.365	0.684	0.506	−66.618 × 10^8^	12.854 × 10^9^
Pre-Radiation	0	0					
Mid-Radiation	−12.462 × 10^7^	82.916 × 10^7^	48.845	−0.150	0.881	−17.910 × 10^8^	15.418 × 10^8^
1-Month Post-Radiation	−11.059 × 10^8^	69.625 × 10^7^	45.806	−1.588	0.119	−25.075 × 10^8^	29.575 × 10^7^
6-Months Post-Radiation	**−26.474×10^8^ ***	70.782 × 10^7^	46.379	−3.740	**0.001 ***	−40.719 × 10^8^	−12.230 × 10^8^
**CD63**							
**Intercept**	25.292 × 10^7^	15.959 × 10^7^	13.187	1.585	0.137	−91.355 × 10^6^	59.720 × 10^7^
Pre-Radiation	0	0					
Mid-Radiation	15.487 × 10^6^	29.691 × 10^6^	46.880	0.522	0.604	−44.248 × 10^6^	75.222 × 10^6^
1-Month Post-Radiation	−2.840 × 10^6^	25.243 × 10^6^	44.809	−0.112	0.911	−53.689 × 10^6^	48.009 × 10^6^
6-Months Post-Radiation	−23.222 × 10^6^	24.822 × 10^6^	44.676	−0.936	0.355	−3.226 × 10^6^	26.782 × 10^6^
**CD64**							
**Intercept**	29.033 × 10^6^	30.066 × 10^6^	11.759	0.966	0.354	−36.624 × 10^6^	94.690 × 10^6^
Pre-Radiation	0	0					
Mid-Radiation	−2.862 × 10^6^	4.926 × 10^6^	46.091	−0.581	0.564	−12.777 × 10^6^	7.052 × 10^6^
1-Month Post-Radiation	−3.362 × 10^6^	3.988 × 10^6^	43.284	−0.843	0.404	−11.402 × 10^6^	4.679 × 10^6^
6-Months Post-Radiation	**−13.391 × 10^6^ ***	3.924 × 10^6^	43.031	−3.413	**0.001 ***	−21.304 × 10^6^	−5.478 × 10^6^
**CD66a**							
**Intercept**	−66.405 × 10^6^	14.097 × 10^7^	3.580	−0.471	0.665	−47.660 × 10^7^	34.379 × 10^7^
Pre-Radiation	0	0					
Mid-Radiation	−95.612 × 10^6^	59.322 × 10^6^	34.123	−1.612	0.116	−21.615 × 10^7^	24.930 × 10^6^
1-Month Post-Radiation	−57.626 × 10^6^	55.213 × 10^6^	56.989	−1.044	0.301	−16.819 × 10^7^	52.937 × 10^6^
6-Months Post-Radiation	−65.729 × 10^6^	54.033 × 10^6^	40.912	−1.216	0.231	−17.486 × 10^7^	43.400 × 10^6^
**H3Cit**							
**Intercept**	88.019 × 10^7^	22.375 × 10^8^	12.802	0.393	0.701	−39.613 × 10^8^	57.217 × 10^8^
Pre-Radiation	0	0					
Mid-Radiation	−35.022 × 10^7^	33.822 × 10^7^	45.854	−1.035	0.306	−10.311 × 10^8^	33.065 × 10^7^
1-Month Post-Radiation	**−95.658 × 10^7^ ***	281.770	43.628	−3.395	**0.001 ***	−15.246 × 10^8^	−38.858 × 10^7^
6-Months Post-Radiation	**−17.000 × 10^8^ ***	29.568 × 10^7^	43.604	−5.750	**0.000 ***	−22.961 × 10^8^	−11.040 × 10^8^

* Statistically Significant at *p* < 0.05. ^a^ Adjustment for multiple comparisons: Least Significant Difference (LSD).

**Table A9:** oPMN marker fold-change and descriptive statistics.

	*N*	Minimum	Maximum	Mean	Std. Deviation
**CD11b Counts**					
Pre-Radiation	67	1.00	1.00	1.0000	0.00000
Mid-Radiation	37	0.23	2.62	0.8644	0.59623
1-Month Post-Radiation	34	0.09	3.48	0.8208	0.66378
6-Months Post-Radiation	25	0.24	1.43	0.5473	0.26527
**CD14 Counts**					
Pre-Radiation	67	1.00	1.00	1.0000	0.00000
Mid-Radiation	32	0.00	5.05	1.0845	0.92528
1-Month Post-Radiation	33	0.00	9.08	1.4551	2.01049
6-Months Post-Radiation	23	−0.33	4.49	1.2296	1.48320
**CD16 Counts**					
Pre-Radiation	68	1.00	1.00	1.0000	0.00000
Mid-Radiation	36	0.33	2.39	0.8335	0.39870
1-Month Post-Radiation	35	0.21	2.10	0.7719	0.41322
6-Months Post-Radiation	26	0.34	1.38	0.7435	0.31551
**CD18 Counts**					
Pre-Radiation	67	1.00	1.00	1.0000	0.00000
Mid-Radiation	36	0.32	4.52	1.0380	0.71241
1-Month Post-Radiation	35	0.30	2.11	0.9364	0.47519
6-Months Post-Radiation	24	0.07	1.32	0.5985	0.33926
**CD63 Counts**					
Pre-Radiation	66	1.00	1.00	1.0000	0.00000
Mid-Radiation	35	0.32	3.34	1.1000	0.58173
1-Month Post-Radiation	33	0.35	2.93	1.1617	0.66603
6-Months Post-Radiation	25	0.45	2.00	0.8897	0.37018
**CD64 Counts**					
Pre-Radiation	64	1.00	1.00	1.0000	0.00000
Mid-Radiation	34	−0.12	8.54	1.1795	1.36600
1-Month Post-Radiation	33	0.34	12.32	1.2803	2.02432
6-Months Post-Radiation	25	0.28	1.65	0.7709	0.37776
**CD66a Counts**					
Pre-Radiation	68	1.00	1.00	1.0000	0.00000
Mid-Radiation	37	0.12	4.90	1.1066	0.89100
1-Month Post-Radiation	35	0.31	8.28	1.3483	1.37298
6-Months Post-Radiation	26	0.06	5.64	1.0697	1.06293
**H3Cit Counts**					
Pre-Radiation	66	1.00	1.00	1.0000	0.00000
Mid-Radiation	35	0.00	22.20	1.8254	3.77952
1-Month Post-Radiation	33	0.00	8.99	0.9346	1.63776
6-Months Post-Radiation	22	−0.01	0.03	0.0066	0.00871

**Table A10:** Statistically significant results of the LMM analyses for oPMN marker fold-changes for CD11b, CD16, 1CD8 CD64 and H3Cit.

Source	Numerator df	Denominator df	F	Sig.
**CD11b Counts**				
Intercept	1	13.007	11.385	0.005
Time	3	46.818	6.945	**0.001 ***
Mucositis	1	53.708	0.262	0.611
Viral Load	1	10.446	0.039	0.847
Sex	1	10.877	0.903	0.363
Smoking Status	2	14.209	0.376	0.693
Alcohol Intake	2	11.043	0.252	0.781
Periodontal Disease	3	10.887	0.948	0.451
Medications Affecting oPMNs	1	12.411	0.026	0.874
Treatment Modality	3	12.404	0.073	0.974
**CD16 Counts**				
Intercept	1	15.283	45.779	0.000
Time	3	50.073	4.035	**0.012 ***
Mucositis	1	55.481	3.646	0.061
Viral Load	1	10.654	0.302	0.594
Sex	1	10.922	3.564	0.086
Smoking Status	2	14.681	0.589	0.567
Alcohol Intake	2	11.308	1.161	0.348
Periodontal Disease	3	10.923	1.545	0.258
Medications Affecting oPMNs	1	13.399	0.164	0.692
Treatment Modality	3	14.049	0.106	0.955
**CD18 Counts**				
Intercept	1	10.239	2.527	0.142
Time	3	45.981	4.224	**0.010 ***
Mucositis	1	54.576	6.927	**0.011 ***
Viral Load	1	7.566	2.876	0.130
Sex	1	7.264	0.342	0.577
Smoking Status	2	9.949	0.193	0.828
Alcohol Intake	2	7.618	0.493	0.629
Periodontal Disease	3	7.359	1.614	0.267
Medications Affecting oPMNs	1	9.092	1.890	0.202
Treatment Modality	3	9.427	0.365	0.780
**CD64 Counts**				
Intercept	1	13.940	10.961	0.005
Time	3	46.030	3.364	**0.026 ***
Mucositis	1	51.313	2.919	0.094
Viral Load	1	10.450	0.135	0.721
Sex	1	10.650	1.272	0.284
Smoking Status	2	13.442	0.054	0.948
Alcohol Intake	2	10.797	0.615	0.559
Periodontal Disease	3	10.739	0.461	0.715
Medications Affecting oPMNs	1	12.991	1.106	0.312
Treatment Modality	3	12.874	0.131	0.940
**H3Cit Counts**				
Intercept	1	9.639	0.001	0.972
Time	3	40.742	7.934	**<0.001 ***
Mucositis	1	49.055	4.913	0.031
Viral Load	1	9.003	0.032	0.863
Sex	1	7.445	1.229	0.302
Smoking Status	2	9.392	0.298	0.749
Alcohol Intake	2	7.868	0.331	0.728
Periodontal Disease	3	7.734	1.306	0.340
Medications Affecting oPMNs	1	8.743	0.935	0.360
Treatment Modality	3	9.086	0.345	0.794

* Statistically significant at *p* < 0.05.

**Table A11:** Pairwise comparisons of the mean difference in the oPMN marker fold-changes.

Time (a)	Time (b)	Adjusted Mean Difference (a–b)	Std. Error	df	Sig. ^a^	95% Confidence Interval	
						Lower Bound	Upper Bound
**CD11b Counts**							
Pre-Radiation	Mid-Radiation	0.177	0.119	48.791	0.141	-0.061	0.416
Pre-Radiation	1-Month Post-Radiation	**0.304 ***	0.100	45.735	**0.004 ***	0.103	0.504
Pre-Radiation	6-Months Post-Radiation	**0.435 ***	0.101	46.459	**<0.001 ***	0.232	0.638
**CD14 Counts**							
Pre-Radiation	Mid-Radiation	1.045	0.577	43.384	0.077	−0.118	2.209
Pre-Radiation	1-Month Post-Radiation	−0.078	0.415	42.422	0.851	−0.916	0.759
Pre-Radiation	6-Months Post-Radiation	−0.148	0.425	42.555	0.730	−1.004	0.709
**CD16 Counts**							
Pre-Radiation	Mid-Radiation	0.079	0.086	52.558	0.363	−0.094	0.252
Pre-Radiation	1-Month Post-Radiation	**0.211 ***	0.073	48.628	**0.006 ***	0.064	0.358
Pre-Radiation	6-Months Post-Radiation	**0.204 ***	0.073	48.662	**0.007 ***	0.058	0.350
**CD18 Counts**							
Pre-Radiation	Mid-Radiation	**−0.391 ***	0.186	49.123	**0.041 ***	−0.765	−0.017
Pre-Radiation	1-Month Post-Radiation	0.086	0.158	43.647	0.588	−0.232	0.405
Pre-Radiation	6-Months Post-Radiation	0.313	0.160	44.715	0.057	−0.010	0.635
**CD63 Counts**							
Pre-Radiation	Mid-Radiation	−0.252	0.147	43.633	0.093	−0.549	0.044
Pre-Radiation	1-Month Post-Radiation	0.073	0.131	41.653	0.580	−0.191	0.337
Pre-Radiation	6-Months Post-Radiation	0.023	0.126	40.672	0.858	−0.231	0.277
**CD64 Counts**							
Pre-Radiation	Mid-Radiation	−0.192	0.131	47.676	0.148	−0.455	0.071
Pre-Radiation	1-Month Post-Radiation	0.056	0.104	45.136	0.597	−0.155	0.266
Pre-Radiation	6-Months Post-Radiation	**0.226 ***	0.102	44.596	**0.032 ***	0.021	0.432
**CD66a Counts**							
Pre-Radiation	Mid-Radiation	−0.261	0.220	49.278	0.241	−0.704	0.181
Pre-Radiation	1-Month Post-Radiation	0.012	0.185	45.936	0.950	−0.361	0.384
Pre-Radiation	6-Months Post-Radiation	−0.069	0.183	45.965	0.710	−0.438	0.301
**H3Cit Counts**							
Pre-Radiation	Mid-Radiation	−0.514	0.272	42.148	0.066	−0.064	0.035
Pre-Radiation	1-Month Post-Radiation	0.314	0.217	40.153	0.156	−0.125	0.753
Pre-Radiation	6-Months Post-Radiation	**0.815 ***	0.236	39.187	**0.001 ***	0.338	1.291

* Statistically Significant at *p* < 0.05. ^a^ Adjustment for multiple comparisons: Least Significant Difference (LSD).

**Table A12:** The fixed effect estimates for the oPMN marker fold-changes.

Parameter	Estimate	Std. Error	df	t	Sig.	95% Confidence Interval	
						Lower Bound	Upper Bound
**CD11b**							
Intercept	0.593	0.649	12.748	0.914	0.377	−0.811	1.998
Pre-Radiation	0	0					
Mid-Radiation	−0.177	0.119	48.791	−1.498	0.141	−0.416	0.061
1-Month Post-Radiation	**−0.304 ***	0.100	45.735	−3.045	**0.004 ***	−0.504	−0.103
6-Months Post-Radiation	**−0.435 ***	0.101	46.459	−4.311	**<0.001 ***	−0.638	−0.232
**CD14**							
**Intercept**	8.472	2.263	13.286	3.744	0.002	3.594	13.350
Pre-Radiation	0	0					
Mid-Radiation	−1.045	0.577	43.384	−1.812	0.077	−2.209	0.118
1-Month Post-Radiation	0.078	0.415	42.422	0.189	0.851	−0.759	0.916
6-Months Post-Radiation	0.148	0.425	42.555	0.348	0.730	−0.709	1.004
**CD16**							
**Intercept**	0.852	0.375	14.904	2.272	0.038	0.052	1.651
Pre-Radiation	0	0					
Mid-Radiation	−0.079	0.086	52.558	−0.918	0.363	−0.252	0.094
1-Month Post-Radiation	**−0.211 ***	0.073	48.628	−2.887	**0.006 ***	−0.358	−0.064
6-Months Post-Radiation	**−0.204 ***	0.073	48.662	−2.810	**0.007 ***	−0.350	−0.058
**CD18**							
**Intercept**	−0.085	0.834	9.971	−0.102	0.921	−1.943	1.774
Pre-Radiation	0	0					
Mid-Radiation	**0.391 ***	0.186	49.123	2.101	**0.041 ***	0.017	0.765
1-Month Post-Radiation	−0.086	0.158	43.647	−0.545	0.588	−0.405	0.232
6-Months Post-Radiation	−0.313	0.160	44.715	−1.950	0.057	−0.635	0.010
**CD63**							
**Intercept**	0.886	0.673	10.931	1.317	0.215	−0.596	2.367
Pre-Radiation	0	0					
Mid-Radiation	0.252	0.147	43.633	1.717	0.093	−0.044	0.549
1-Month Post-Radiation	−0.073	0.131	41.653	−0.558	0.580	−0.337	0.191
6-Months Post-Radiation	−0.023	0.126	40.672	−0.181	0.858	−0.277	0.231
**CD64**							
**Intercept**	0.511	0.592	13.472	0.864	0.403	−0.764	1.787
Pre-Radiation	0	0					
Mid-Radiation	0.192	0.131	47.676	1.470	0.148	−0.071	0.455
1-Month Post-Radiation	−0.056	0.104	45.136	−0.533	0.597	−0.266	0.155
6-Months Post-Radiation	**−0.226 ***	0.102	44.596	−2.218	**0.032 ***	−0.432	−0.021
**CD66a**							
**Intercept**	0.650	1.194	12.357	0.544	0.596	−1.943	3.243
Pre-Radiation	0	0					
Mid-Radiation	0.261	0.220	49.278	1.186	0.241	−0.181	0.704
1-Month Post-Radiation	−0.012	0.185	45.936	−0.063	0.950	−0.384	0.361
6-Months Post-Radiation	0.069	0.183	45.965	0.374	0.710	−0.301	0.438
**H3Cit**							
Intercept	−1.181	1.340	9.732	−0.881	0.399	−4.178	1.816
Pre-Radiation	0	0					
Mid-Radiation	0.514	0.272	42.148	1.888	0.066	−0.035	1.064
1-Month Post-Radiation	−0.314	0.217	40.153	−1.447	0.156	−0.753	0.125
6-Months Post-Radiation	**−** **0.815 ***	0.236	39.187	−3.459	**0.001 ***	−1.291	−0.338

* Statistically Significant at *p* < 0.05.
